# The effect of clinical information on radiology reporting: A systematic review

**DOI:** 10.1002/jmrs.424

**Published:** 2020-09-01

**Authors:** Chelsea Castillo, Tom Steffens, Lawrence Sim, Liam Caffery

**Affiliations:** ^1^ Centre for Online Health The University of Queensland Brisbane QLD Australia; ^2^ Department of Diagnostic Radiology Princess Alexandra Hospital Brisbane QLD Australia

**Keywords:** Radiology, report, request, clinical information, communication

## Abstract

**Introduction:**

The aim of this study was to investigate the effects of clinical information on the accuracy, timeliness, reporting confidence and clinical relevance of the radiology report.

**Methods:**

A systematic review of studies that investigated a link between primary communication of clinical information to the radiologist and the resultant report was conducted. Relevant studies were identified by a comprehensive search of electronic databases (PubMed, Scopus and EMBASE). Studies were screened using pre‐defined criteria. Methodological quality was assessed using the Joanna Briggs Institute (JBI) Critical Appraisal Checklist for Quasi‐Experimental Studies. Synthesis of findings was narrative. Results were reported according to the Preferred Reporting Items for Systematic Reviews and Meta‐Analyses (PRISMA) guidelines.

**Results:**

There were 21 studies which met the inclusion criteria, of which 20 were included in our review following quality assessment. Sixteen studies investigated the effect of clinical information on reporting accuracy, three studies investigated the effect of clinical information on reporting confidence, three studies explored the impact of clinical information on clinical relevance, and two studies investigated the impact of clinical information on reporting timeliness. Some studies explored multiple outcomes. Studies concluded that clinical information improved interpretation accuracy, clinical relevance and reporting confidence; however, reporting time was not substantially affected by the addition of clinical information.

**Conclusion:**

The findings of this review suggest clinical information has a positive impact on the radiology report. It is in the best interests of radiologists to communicate the importance of clinical information to reporting via the creation of criteria standards to guide the requesting practices of medical imaging referrers. Further work is recommended to establish these criteria standards.

## Graphical abstract

The aim of this review was to investigate the effects of clinical information on the radiology report. We conducted a systematic review of studies that investigated a link between primary communication of clinical information to the reporting radiologist and the resultant report. The findings of this review suggest that clinical information is beneficial to radiology reporting. It is in the best interests of radiologists to communicate the importance of clinical information on reporting via the creation of criteria standards to guide the requesting practices of medical imaging referrers. Further work is recommended to establish these criteria standards.

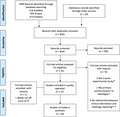

## Introduction

It is common practice for radiologists to interpret imaging examinations and formulate a report using clinical information communicated to assist with this process. Clinical information refers to all information detailing the patient's clinical situation and can include the current problem, co‐existing and past medical history, current medications, allergies, fasting status, suspected diagnosis and clinical question to be answered.^1^ It is used to provide the radiologist with a greater understanding of the clinical context.

For all medical imaging examinations in Australia to be performed, a request must be completed by a referrer.^2^, ^3^ The request must list the patient's identifying details and indicate the type of examination requested.^2^, ^3^ It is also essential that the referrer provides adequate clinical information describing the reason for the examination.^1^ The request must be signed and dated by the referrer.^2^ This allows compliance with radiation safety regulations and maximum workflow efficiency.

When the patient presents to the referrer, they are medically assessed and a request for imaging is completed, using information about the patient's medical history and current presentation. This request can take one of two paths from the referrer to the radiologist, via the radiographer, who completes the imaging before sending it along with the request to the radiologist; or the request is transmitted directly to the radiologist who then reviews the clinical information and selects the imaging protocol to be performed, before transferring it to the radiographer. The radiologist is also able to review clinical information in the request when interpreting imaging and formulating their report.

Loy & Irwig's^4^ 2004 review established that radiology reporting with clinical information improved interpretation accuracy. Since this review, there have been technological advances such as the increased use of cross‐sectional imaging and widespread adoption of electronic health records (EHR). These developments may have reduced the referring clinician's perception of the importance of clinical information on radiology reporting, as it may be assumed that this clinical information is readily available and easily accessed by all clinicians and medical imaging staff.^5^ The aim of this study was to investigate the effects of clinical information communicated to the radiologist, on the accuracy, timeliness, reporting confidence and clinical relevance of the radiology report.

## Methods

### Search strategy

This review followed the methods described in a published protocol in the PROSPERO register (CRD42019138509).^6^ To identify relevant articles the PubMed, Scopus and EMBASE databases were searched using relevant keywords for request, clinical information, diagnostic imaging and radiology report. The syntax used to search the PubMed electronic database is detailed in Table 1. No limits were placed on publication date. Searches were conducted in June 2019.

**Table 1:** Search syntax for PubMed database.

Database	Syntax
PubMed	((((request[Title/Abstract] OR requests[Title/Abstract] OR referral[Title/Abstract] OR referrals[Title/Abstract] OR requisition[Title/Abstract] OR requisitions[Title/Abstract] OR order[Title/Abstract] OR orders[Title/Abstract])) AND (“clinical information”[Title/Abstract] OR “clinical detail*”[Title/Abstract] OR “clinical history”[Title/Abstract] OR “clinical value”[Title/Abstract] OR “clinical indication*”[Title/Abstract] OR “patient data”[Title/Abstract] OR “patient information”[Title/Abstract] OR “patient history”[Title/Abstract] OR symptom*[Title/Abstract] OR "clinical question*"[Title/Abstract] OR "clinical sign*”[Title/Abstract])) AND (ct[Title/Abstract] OR “ct scan”[Title/Abstract] OR “computerized tomography”[Title/Abstract] OR “computed tomography”[Title/Abstract] OR radiology[Title/Abstract] OR “diagnostic imaging”[Title/Abstract] OR “medical imaging”[Title/Abstract] OR radiography[Title/Abstract] OR x‐ray[Title/Abstract] OR "magnetic resonance imaging”[Title/Abstract] OR mri[Title/Abstract] OR mammography[Title/Abstract] OR ultrasound[Title/Abstract] OR sonography[Title/Abstract])) AND (“radiology report*”[Title/Abstract] OR “diagnostic report*”[Title/Abstract] OR “clinical report*”[Title/Abstract] OR interpretation[Title/Abstract])

### Inclusion and exclusion criteria

Studies were included if they were as follows: (1) primary studies, published in peer‐reviewed journals, (2) related to diagnostic imaging for any population of human patients and (3) investigated a relationship between primary communication of clinical information to the radiologist and the resultant radiology report. This review defined primary communication as any method of communication given directly to the radiologist, such as clinical information accompanying imaging (within the medical imaging request and additional information provided at the time of imaging), clinical information received in patient charts or verbal communication between referrer and radiologist. Studies published in languages other than English were excluded. Conference proceedings, reviews, case reports, study protocols, commentary and letters to the editor were also excluded.

### Selection process

After duplicates were removed, titles and abstracts of studies were screened by two reviewers (CC and TS) to determine eligibility for inclusion. Screening of full text of publications was performed if the abstract provided insufficient information to judge eligibility. Disagreement or uncertainty of study eligibility was resolved by consensus discussion. The reference lists of all included studies were interrogated and subjected to the same screening process.

### Data extraction and quality assessment

The full text of included studies was read by two reviewers (CC and LC). Data were extracted on study characteristics (year, diagnostic test/s, indications or disease, reference standard, number of studies, number of reviewers, methodology), interobserver agreement, outcome measures and results summary related to the research question. Data extraction was performed by one reviewer (CC), with validation by a second reviewer (LC). Disagreements were resolved through discussion.

The Joanna Briggs Institute (JBI) Critical Appraisal Checklist for Quasi‐Experimental Studies^7^ was used to assess the quality of each study by examining the extent to which a study addressed the possibility of bias in its design, conduct and analysis. The JBI quality score was a value out of nine points, with higher scores indicating higher quality studies. This checklist included nine questions which assessed internal validity, similarity of participants of compared groups, reliability of outcomes measured and appropriateness of statistical analysis. The quality and risk of bias assessment was conducted independently by two reviewers (CC and LC); disputes were resolved by consensus discussion. A cut‐off score of three was used to exclude low‐quality studies from synthesis.

### Analysis

Whilst some included studies shared commonalities in design, heterogeneity of methodologies, interventions and statistical analysis rendered them difficult to compare statistically. Therefore, a narrative synthesis was conducted to contextualise findings relevant to the review question, these being reporting accuracy, confidence, timeliness and clinical relevance.

The data extraction process allowed us to categorise study characteristics into consistent fields across included studies. The data extraction and categorisation facilitated narrative synthesis by allowing us to examine the context of each study. All authors met regularly during the process and using the extracted data, discussed and subsequently refined the narrative. Results were reported according to the Preferred Reporting Items for Systematic Reviews and Meta‐Analyses (PRISMA) guidelines.^9^

## Results

**Figure 1:**
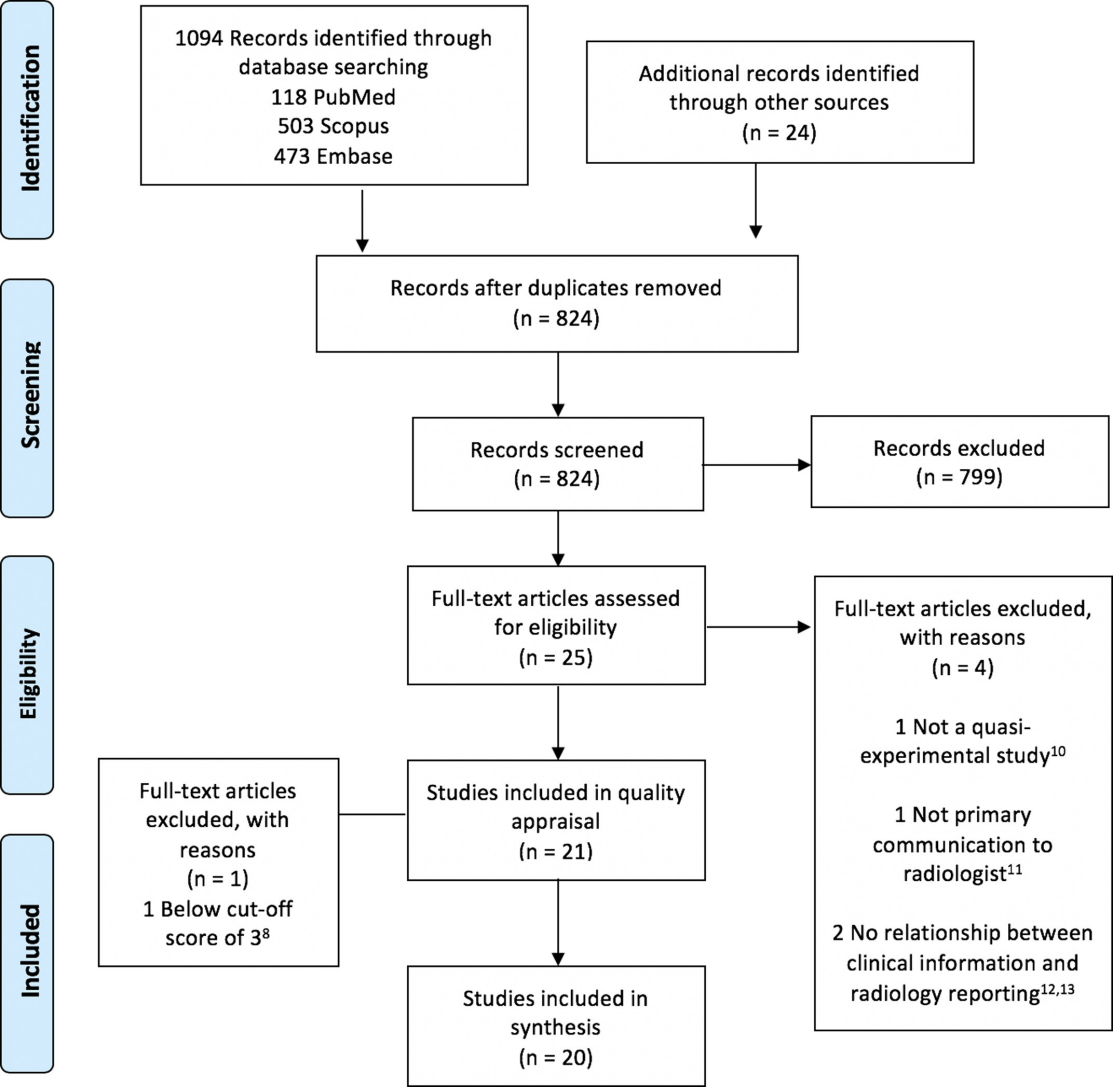
Preferred Reporting Items for Systematic Reviews and Meta‐Analyses (PRISMA) flow diagram.

We identified 21 studies that met our inclusion criteria, and after quality assessment, 20 studies were included in our review. The excluded study^8^ was deemed to lack clarity regarding cause and effect and to have measured outcomes in an unreliable way. The results for each stage of the search are demonstrated in the PRISMA flow diagram^9^ (Fig. 1).

### Study characteristics

Sixteen studies^14^, ^15^, ^16^, ^17^, ^18^, ^19^, ^20^, ^21^, ^22^, ^23^, ^24^, ^25^, ^26^, ^27^, ^28^, ^29^ investigated the effect of clinical information on report accuracy, three studies^16^, ^25^, ^30^ investigated the effect of clinical information on reporting confidence, three studies^32^, ^33^, ^34^ explored the impact of clinical information on clinical relevance, and two studies^24^, ^31^ investigated the impact of clinical information on reporting time. We found three studies^16^, ^24^, ^25^ which investigated the effect of clinical information on more than one outcome. One study^16^ investigated effects on reporting accuracy, confidence and timeliness. Another study^24^ evaluated effects on both reporting accuracy and timeliness, and another explored the effects on both reporting accuracy and confidence.^25^

X‐ray examinations were the diagnostic test in 12 (57%) of included studies.^8^, ^16^, ^20^, ^21^, ^22^, ^23^, ^24^, ^25^, ^26^, ^28^, ^29^, ^32^ Five studies^18^, ^19^, ^27^, ^30^, ^31^ (24%) focused on computed tomography (CT) and one^33^ (5%) on magnetic resonance imaging (MRI). The remaining three (14%) studies^14^, ^15^, ^17^ included two modalities. The X‐ray studies were published between 1963 and 2014. Six of 12 studies^8^, ^21^, ^24^, ^26^, ^28^, ^29^ focused on chest X‐ray examinations, the remaining five involved chest and abdomen^20^, abdomen^32^, extremity ^16^, ^22^, ^25^ or a combination of X‐ray examinations.^23^ Three of these studies involved paediatric cohorts only.^20^, ^21^, ^32^ Of the five studies ^18^, ^19^, ^27^, ^30^, ^31^ on CT examinations, two^19^, ^27^ focused on CT head, one^30^ on CT abdomen/pelvis, one^31^ on CT temporal bones and one on various^18^ CT scans. These studies were published between 1983 and 2017. The study^33^ on MRI examinations, published in 2010, focused on MRI cervical spine examinations. Of the three studies^14^, ^15^, ^17^ involving examinations of two modalities, two^15^, ^17^ involved CT and MRI and one^14^ X‐ray and ultrasound. These studies were published between 2002 and 2019.

The size of data sets and the number and consistency of reviewers varied throughout studies. Data set sizes ranged from seven^28^ to 561^17^ cases. The number of reviewers ranged from one^32^ to 11.^29^ Some studies featured consistency of readers before and after intervention, whilst others utilised radiologists on duty at the time of reporting and did not disclose the exact number of assessors.

A total of 16 of 20 studies used a similar method involving a sample set of images, assessed twice by a group of reviewers.^8^, ^15^, ^16^, ^18^, ^19^, ^20^, ^22^, ^23^, ^24^, ^25^, ^26^, ^27^, ^28^, ^29^ Each review had different amounts or qualities of clinical information. Three studies^14^, ^17^, ^32^ asked radiologists to subjectively rate the impact of available clinical information on reporting, and one study^31^ evaluated the impact of clinical information in two samples, pre‐ and post‐intervention. This study was one of two which featured departmental guidelines to classify clinical information in requests as either adequate or inadequate. One study^17^ evaluated the impact of clinical indications of stroke in CT head and MRI brain requests on final discharge diagnosis. The other CT and MRI study^15^ compared clinical information in imaging requests with clinical information available to the referrer at the time of requesting. The remaining study^14^ involving X‐ray and ultrasound evaluated the impact of additional clinical information contributed by imaging technologists on the quality of the report. This study instructed imaging technologists to contribute clinical information on patient symptoms, including duration and onset.

Additional information available to readers varied significantly between studies. Whilst many included all clinical information available to referrers at the time of reporting in the second read, others tried to demonstrate effect of an intervention to evaluate any change to reporting. These interventions included patient questionnaires,^30^ inclusion of a clinical question,^33^ additional information from imaging technologists^14^ and a graphic indicating site of pain.^16^ The results of the data extraction from the included studies are shown in Table 2.

**Table 2:** Data extraction.

Source	Diagnostic test/indication	Studies/readers	Method	Info – first review	Additional info – subsequent review	Relevant findings	JBI (n/9)
Maizlin & Somers, 2019^14^	XR and US/ ++	250/Multiple	Radiologists characterised the effect of additional info on imaging interp.	Original imaging request	Clinical info from imaging techs	Added info deemed important in 173 cases (69.2%), not critically important in 77 cases (30.8%). Significantly more useful for radiographic examinations compared to ultrasound.	3
Lacson et al, 2018^15^	MRI L‐spine and CT AP/ ++	315/4	Same studies reviewed twice by the same readers	Original imaging request	Provider notes (medical Hx, physical examination, progress, phone notes)	Potential impact of incomplete or inaccurate order indications on examination interpretation in 135/315 cases (43%). IOA = moderate to strong agreement ‐ discordance (K = 0.89), incompleteness (K = 0.72).	6
Doshi et al, 2017^30^	CT Abdo + Pelvis/ Abdo pain	100/2	Same studies reviewed twice (6 weeks apart) by the same readers	Original imaging request	Patient questionnaire – current Sx, previous surgery, localisation of pain	R1: Cause of pain identified in 7 cases post‐questionnaire (37% increase). Confidence 4.8 ± 0.6. R2: Cause of pain identified in 4 cases post‐questionnaire (16% increase). Confidence 4.9 ± 0.3	4
Qureishi et al, 2014^31^	CT Temporal Bones/ Various	100/2	2 samples evaluated by 2 assessors, pre‐ and post‐intervention against departmental guideline	Request and report (pre‐intervention)	Request and report (post‐intervention)	Post‐intervention ‐ percentage of reports indicating a Dx or excluding an important complication increased (52 to 94%).	7
Sarwar et al, 2014^16^	XR Foot/ Subtle foot Fx	226/7	Same studies reviewed twice (6 months apart) by the same readers	Text Hx	Graphic indicating site of pain	Accuracy (79 to 82%). Sensitivity ‐ Fx detection (67 to 73%). Degree of confidence (8.1 to 8.4). Interp time (53 to 50 sec). Specificity (93% to 94%).	6
Cohen & Ellett, 2012^32^	XR Paed Abdo/ NGT position	188/1	Reports reviewed for quality – with and without addition of clinical question	Original imaging request	CQ: ‘newly placed NGT for evaluation of tube tip position’	CQ answered in 95% of cases when specifically asked (134/141 studies). When the request failed to pose clinical question, pertinent info (tube location) was mentioned in 31% (*n* = 4) of cases.	3
Aubin et al, 2010^33^	MRI C‐Spine/ VA path	79/6	Same studies reviewed twice by the same readers	Patient Sx	CQ	Pathology described (0%) in any cases where CQ was not posed. Pathology described in 100% of cases where CQ was posed.	6
Mullins et al, 2002^17^	CT Head and MRI Brain/ Stroke	561 CT, 409 MRI/ ++	CT and MRI report results compared with the final discharge Dx	Original imaging request	Radiology report and discharge Dx	Accuracy of stroke detection on CT higher when pertinent clinical info in the request (59% vs 47%). No statistically different outcomes in accuracy of stroke detection on MRI vs CT(94% vs 95%).	4
Leslie, Jones & Goddard, 2000^18^	CT/ ++	50 cases, 100 reports/3	Same studies reviewed twice by 2 of 3 same readers. Each CT examination double reported	Name, age, sex of patient	Original imaging request	19/100 reports changed after clinical info known. More accurate reports in 67% (*n* = 10) of cases. Less accurate reports in 3 of 5 cases where the clinical info in the request was incorrect. IOA = 60% agreement. Weak agreement between readers’ change in opinion and added clinical info (K = 0.42)	4
Berbaum et al, 1994^20^	Paed XR Chest and Abdo/ ++	64/9	Same studies reviewed twice by the same readers (4 months apart), 3 reads per study	Patient age and sex	i) Clinical Hx provided before study viewed, ii) clinical Hx provided after study viewed	Appropriate Hx given before viewing study, accuracy was greater than with the same Hx provided after viewing study (.745 vs .693, *P* < 0.01) or without history (.745 vs 0675, *P* < 0.01). No increase in detection accuracy with hx provided after inspection than without Hx (.693 vs .675, *P* > 0.05).	5
Babcook, Norman & Coblentz, 1993^21^	Paed XR Chest/ Bronchiolitis	50/4	Equivocal studies only read twice by the same readers	Consistent clinical Hx (+ve XR/+ve Hx and ‐ve XR/‐ve Hx)	Inconsistent clinical Hx (+ve XR/‐ve Hx and ‐ve XR/ +ve Hx)	Significantly more features identified on the equivocal normal XRs when assigned a + ve clinical Hx. No significant difference in the number of features identified on the equivocal bronchiolitis XRs, regardless of the clinical Hx	3
Rickett, Finlay & Jagger, 1992^22^	XR Extremity (Trauma)/ Subtle Fx or dislocation	50/7	Same studies reviewed twice by the same readers	Simple description of ROI (e.g. injured hand)	Complete anatomical localisation of symptoms	Diagnostic accuracy was improved from 253 (72.3%) to 281 (80.3%) when localisation clues provided (highly significant). Fx Dx improved by 60%. The accuracy of all but one reader improved with localisation clues. All readers had fewer false negatives.	6
Song et al, 1992^23^	XR/ Various	109/8	Same studies reviewed twice (1 month apart) by the same readers	Without clinical Hx	Original imaging request and patient chart	The mean areas under the ROC curves without and with clinical history were 0.75+/‐0.12 and 0.84+/‐0.08, respectively (stat sig). Knowledge of clinical history improved diagnostic accuracy for readers of various experience levels	4
Cooperstein et al, 1990^24^	XR Chest/ Various	247/5	Same studies reviewed twice by the same readers. Reference standard created by group of 20 radiologists	Without clinical Hx	With clinical history (as detailed by request requirements)	No significant differences in readers’ performance between interpretations made with or without history. Average additional time of 6.5sec needed when interpreting with clinical history.	5
Berbaum et al, 1988^25^	XR Extremity/ Subtle Fx	40/7	Same studies reviewed twice (4 months apart) by the same readers.	No location‐specific clinical hx	Location‐specific clinical hx	Interpretations with location‐specific hx were significantly more accurate than without.	6
Berbaum et al, 1988^26^	XR Chest/ Nodules/lesions	44/6	Same studies reviewed twice (++ months apart) by the same readers.	Patient age and sex	Tentative diagnosis	Tentative diagnosis improves detection of more complex lesions, but not of simple nodules.	4
Berbaum et al, 1986^8^	XR Chest/ ++	43/6	Same studies reviewed 3 times (++ months apart) by the same readers.	Patient age and sex	Tentative diagnosis	Provision of tentative Dx resulted in significantly greater accuracy than without.	2
McNeil et al, 1983^27^	CT Head/ ++	89/4	Same studies reviewed twice (2 weeks apart) by the same readers	Patient age and sex	All clinical info available at the time the study was requested	The inclusion of clinical hx resulted in 3.3% increase in accuracy of interpretation.	6
Doubilet & Herman, 1981^28^	XR Chest/ Various	7 cases, 8 abnorm/ ++	Same studies reviewed twice by ++ readers.	Unrelated clinical Hx	Clinical hx suggestive of abnormality	True‐positive rate increased from 38% (non‐suggestive hx) to 84% (suggestive hx), a statistically significant increase. All false positives (*n* = 9) were suggested by the clinical hx.	4
von Kummer et al, 1996^19^	CT Head/ Cerebral ischaemic infarction	45/6	Same studies reviewed twice (30mins apart) by the same readers	Knowledge studies were from stroke pop'n, blinded to side of Sx	Knowledge of side of hemiparesis based on clinical signs and symptoms	No significant difference in blinded and unblinded results. The blinded observer may misinterpret signs of infarction in up to 31% of scans. IO A = varied: Overall – (49% to 71%). Between radiologists and reference radiologist – (69% to 93%)	5
Schreiber, 1963^29^	XR Chest/ ++	100/11	Same studies read twice (4 months apart) by the same readers	Without clinical Hx	Clinical hx	Interpretations done with clinical hx showed significantly more correct readings.	4

Abbreviations: Abdo = abdomen; abnorm = abnormalities; CQ = clinical question; CT = computed tomography; CT AP = computed tomography of abdomen and pelvis; Dx = diagnosis; exam/s = examination/s; Fx = fracture/s; Hx = history; info = information; interp = interpretation; IOA = interobserver agreement; JBI/9 = score (out of 9) from Joanna Briggs’ Institute study appraisal tool; MRI = magnetic resonance imaging; NGT = nasogastric tube; paed = paediatric; path = pathology; pop'n = population R = reader; ref standard = reference standard; ROI = region of interest; sec = seconds; stat sig = statistically significant; Sx = symptoms; tech/s = technologist/s; US = ultrasound; VA = vertebral artery; XR/s = radiograph/s; ‐ve = negative; +ve = positive; ++ = multiple

### Study quality

**Figure 2:**
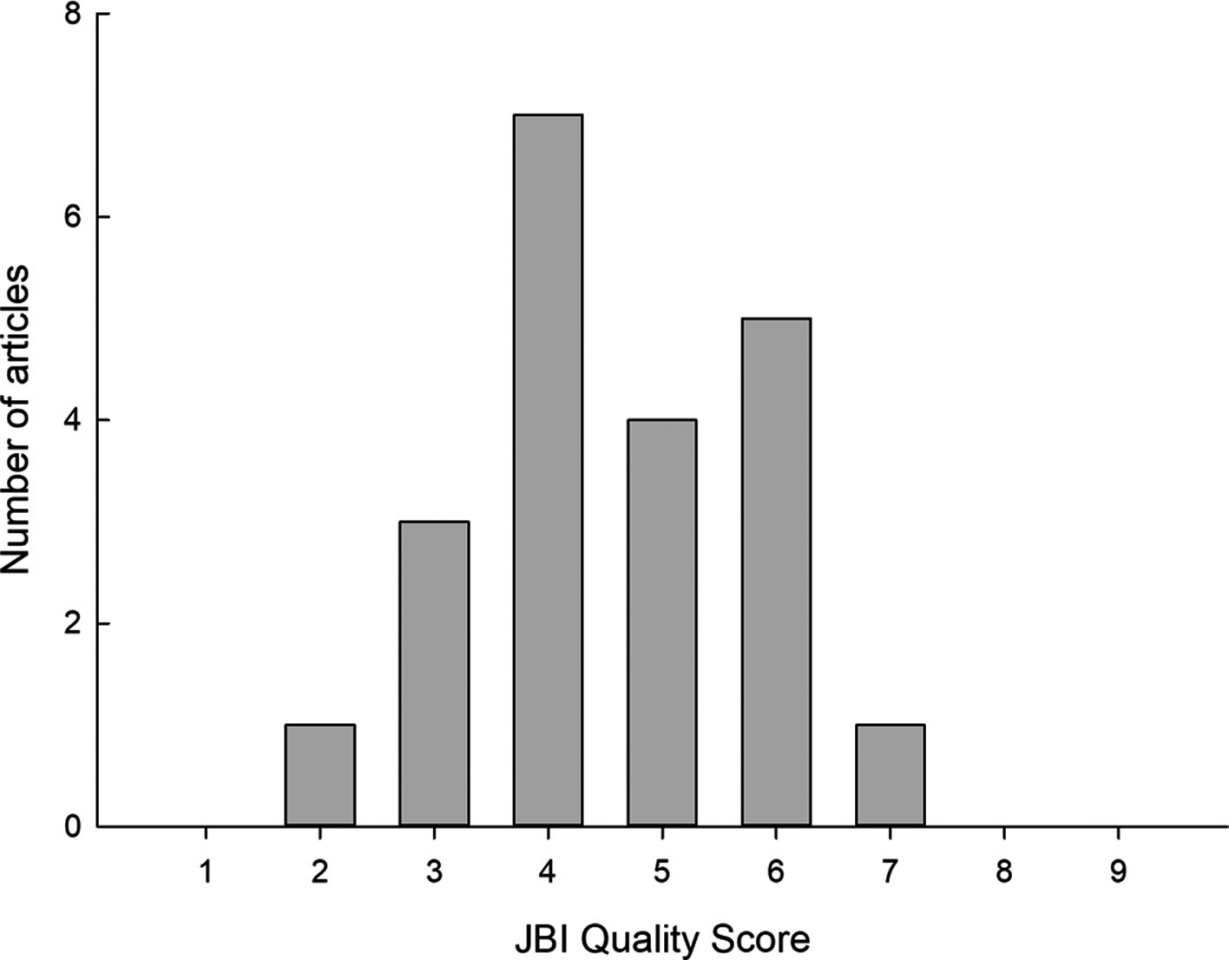
JBI quality and risk of bias assessment scores.

The JBI quality score ranged from 2 to 7 out of a possible 9 points with a median score of 4 (Fig. 2). The highest scoring study was the only study^31^ to include a control group. Lower scores were due to using multiple different assessors instead of one group of assessors, using only one assessor and failure to conduct appropriate statistical analysis.

### Interpretation accuracy

Sixteen studies investigated the effect of clinical information on the accuracy of reporting. Of these, three studies^16^, ^17^, ^28^ reported sensitivity and specificity. All three reported that the addition of clinical information improved sensitivity. Reported changes in sensitivity were 38% to 84%,^28^ 67% to 73%^16^ and 38% to 52%.^17^ Sarwar et al^16^ and Mullins et al^17^ demonstrated improved specificity, whilst Doubilet & Herman^28^ did not. Sarwar et al^16^ reported a change in specificity from 93% to 94%, and Mullins et al^17^ reported an 89% to 96% increase in specificity for CT studies and 95% to 98% for MRI studies.

Six studies used area under the receiver operator characteristic (ROC) curves to quantify the average difference in improvement in accuracy. Results ranged from minimal improvement^20^ to significant improvement.^21^, ^23^, ^25^, ^26^, ^27^ Overall, these studies demonstrated that clinical information improved diagnostic accuracy in various conditions.

Three studies described an impact on overall accuracy of reporting.^17^, ^22^, ^29^ Rickett, Finlay and Jagger^22^ found an increase from 72% to 80% in diagnostic accuracy, Schreiber^29^ reported an improvement in accuracy without numerical data, and Mullins^17^ found an overall improvement in diagnostic accuracy from 47% to 59%.

Three studies described accuracy in terms of influencing change to the original radiologist report. ^14^, ^15^, ^18^ Lacson et al^15^ found 43%, and similarly, Leslie et al^18^ found 38% of reports were changed when clinical information was known. Leslie et al^18^ reported the majority of changes to reports increased accuracy. Maizlin & Somers^14^ determined clinical information to be important for 69% of cases and not critically important for 31%.

Two studies^19^, ^24^ found the addition of clinical information did not change reporting accuracy. The results relevant to the accuracy outcome measure have been further summarised in Table 3.

**Table 3:** Results relevant to accuracy.

Study	Significance test	Significance level	*P*‐value	Authors’ conclusions
Maizlin & Somers, 2019^14^	Chi‐square test, Fisher's exact test	<0.05	Not stated for this outcome	The role of technologist notes for interpreting an examination was deemed important in 173 cases (69.2%) and not critically important in 77 cases (30.8%)
Lacson et al, 2018^15^	Chi‐square test	<0.05	Not stated for outcome of interest	Radiological interpretation was potentially impacted in 43% (135/315) of examinations with incomplete or discordant requests
Sarwar et al, 2014^16^	McNemar's test with Edwards continuity correction	<0.05	*P* (sensitivity) = <0.001, *P* (specificity) = 0.33	Use of graphic increased sensitivity for the presence and absence of subtle fracture from 67% to 73%. Specificity changed from 93% without graphic to 94% with graphic.
Mullins et al, 2002^17^	Fisher's exact two‐tailed test	<0.05	*P* (CT sensitivity) = 0.008, *P* (CT specificity) = 0.680 *P* (MRI sensitivity (0.82), *P* (MRI specificity = 0.528)	For CT, sensitivity for stroke detection was greatest (52%) for stroke group and lowest (38%) for no‐stroke group. Specificity was greater for stroke group (96%) than for no‐stroke group (89%). Overall diagnostic accuracy was higher in stroke group (59% vs 47% in no‐stroke group). For MRI, sensitivity for stroke detection was similar for both groups (95% vs 94%). Specificity was also similar for both groups (95% vs 98%).
Leslie et al, 2000^18^	Kappa coefficient	95% Confidence Interval	19 reports changed after clinical information was known (k = 0.42)	The more complex the investigation, the more important the clinical information. The kappa score of 0.42 indicates clinical information influences different readers in a similar way.
Von Kummer et al, 1996^19^	Kappa coefficient	Not stated	Not stated	Unblinding to clinical question did not affect agreement rates between radiologists.
Berbaum et al, 1994^20^	Receiver operator characteristic (ROC) curve, analysis of variance (ANOVA)	<0.01	With Hx before viewing study, accuracy was greater than with the same Hx provided after viewing study (.745 vs .693, *P* < 0.01) or without history (.745 vs 0675, *P* < 0.01). No increase in detection accuracy with Hx provided after inspection than without history (.693 vs .675, *P*> 0.05).	Clinical history supports abnormality detection accuracy in paediatric chest and abdomen X‐rays when it is considered prior to reading the examination.
Babcook et al, 1993^21^	ROC curve, chi‐square test	<0.05	P = <0.05 (for suggestive history of bronchiolitis, hyperinflation and consolidation)	Radiologists more frequently reported the presence of features on equivocal radiographs accompanied by suggestive history than a non‐suggestive history. In the order of 25‐50%. ROC curves showed overall increase in false‐positive rate, with slight decrease in overall performance.
Rickett et al, 1992^22^	McNemar's test	<0.01	Diagnostic accuracy was improved from 253 (72.3%) to 281 (80.3%) when localisation clues were available. p < 0.00012	Diagnostic accuracy of trauma extremity X‐rays improves when accurate clinical information including injury localisation is provided.
Song et al, 1992^23^	ROC curve, ANOVA, paired t‐tests	0.05	All radiologists, the mean areas under the ROC curves without and with clinical history were 0.75+/‐0.12 and 0.84+/‐0.08, respectively, p < 0.02	Knowledge of clinical history improves diagnostic accuracy for radiologists of various levels of knowledge
Cooperstein et al, 1989^24^	Paired Student's t‐test	<0.05	For disease‐specific comparisons, there was no significant change demonstrated in the results of all readers for any of the abnormalities (p> 0.35)	General clinical history does not support improved accuracy of reporting for specific diseases (interstitial disease, lung nodule, pneumothorax)
Berbaum et al, 1988^25^	ROC curve, ANOVA, paired t‐tests	Not stated	Greater confidence in rating abnormal cases, p = 0.031	Specific clinical information, such as localisation of injury clues improve the ability of radiologists to detect fractures in the trauma patient.
Berbaum et al, 1988^26^	ROC curve, one‐way analysis of variance, Tukey's test.	<0.1	p = <0.1	Clinical history improves detection of diverse, subtle lesions but not of simple nodules.
McNeil et al, 1983^27^	ROC curve	<0.05	p = <0.05	Clinical history significantly improves the interpretation of CT head studies
Doubilet & Herman, 1981^28^	Paired Student's t‐test, Wilcoxon rank‐sum test	<0.01	True‐positive rate increased from 38% (non‐suggestive history) to 84% (suggestive history) p < 0.01	A suggestive clinical history increases the sensitivity and seems to decrease the specificity of interpretation of chest X‐rays. A relevant clinical history increases true‐positive rate of chest X‐rays containing subtle but unambiguous findings.
Schreiber, 1963^29^	Student's t‐test	<0.03	t = 2.65, p = 0.03	Film interpretations done with clinical history provided demonstrated significantly more correct readings than those without clinical history.

### Reporting confidence

Three studies investigated the effect of clinical information on the confidence of reporting, each in a different way.^16^, ^25^, ^30^ Sarwar et al^16^ used a graphic indicating site of maximal pain to complement the request; Berbaum et al^25^ investigated the effect of providing the specific site of injury; Doshi et al^30^ used a patient questionnaire to complement the request; all three reported a positive impact of clinical information on reporting confidence. Sarwar et al^16^ reported an increase in radiologist confidence from 8.1 to 8.4 (on a 10‐point scale), Berbaum et al^25^ concluded that confidence improved without quantifying the improvement, and Doshi et al^30^ found confidence in interpretation to be significantly greater when patient questionnaires were accessed. The results relevant to the reporting confidence outcome measure have been further summarised in Table 4.

**Table 4:** Results relevant to reporting confidence and timeliness.

Study	Outcome measure	Significance test	Significance level	*P*‐value	Authors’ conclusions
Doshi et al, 2017^30^	Reporting confidence	Paired Wilcoxon test	Not reported	*P* < 0.001 for both reader 1 and reader 2	Interpretation confidence significantly greater when patient questionnaire accessed (reader 1: 4.8 ± 0.6 vs. 4.0 ± 0.5; reader 2: 4.9 ± 0.3 vs. 4.7 ± 0.5, *P* < 0.001)
Sarwar et al, 2014^16^	Reporting confidence	Paired Student's *t*‐test, Wilcoxon signed‐rank test	0.05	Improved degree of confidence from 8.1 to 8.4 (*P* < 0.0001)	When radiologists are provided with a graphic, degree of confidence is increased. This may lead to a decrease in hedging, vague reports and unnecessary follow‐up imaging.
Berbaum et al, 1988^25^	Reporting confidence	ROC curve, ANOVA, paired *t*‐test	Not reported	Greater confidence in rating abnormal cases, *P* = 0.031	Localisation clues (within clinical information) improve the ability of radiologists to detect fractures in the trauma patient.
Sarwar et al, 2014^16^	Reporting timeliness	Paired Student's *t*‐test and Wilcoxon signed‐rank test	0.05	Decreased mean interpretation time 6% (*P* = 0.006)	Radiologists require less time for interpretation when the patient's clinical history is complemented by a graphic highlighting the site of maximal pain
Cooperstein et al, 1990^24^	Reporting timeliness	Not reported	Not reported	Not reported	Time needed to display, review, interpret and rate the cases varied only slightly between the two reading environments (with/without clinical information)

### Clinical relevance of reports

The importance of the inclusion of a specific clinical question in the imaging request was investigated in three of the included studies. Aubin et al^34^ focused on characteristics of vertebral arteries on MRI cervical spine requests, Cohen & Ellett^33^ looked at the location of nasogastric (NG) tubes in paediatric chest and abdomen X‐rays, and Qureishi et al^31^ investigated the impact of the inclusion of a clinical question on clinical relevance of CT temporal bone reports. Improvement was demonstrated in all three studies: Aubin et al^34^ from 0% to 100%, Cohen & Ellett^33^ from 31% to 95% and Qureishi et al^31^ from 52% to 94%. The results relevant to the clinical relevance outcome measure have been further summarised in Table 5.

**Table 5:** Results pertaining to clinical relevance.

Study	Significance Test	Result	Authors’ conclusions
Qureishi et al, 2014^31^	Two proportion Z‐test	Percentage of temporal bone CT reports indicating a diagnosis or excluding an important complication increased from 52 to 94 (*P* < 0.01)	The increase in information provided in requests which adhered to departmental guidelines, influenced the improvement in clinical relevance of the report
Cohen & Ellett, 2012^32^	Not reported	When the request indicated tube placement, the location of the tube tip included in the report 134/141 (95%) and not mentioned 7/141 (5%) times. When the request failed to mention tube location within study indication, the report only mentioned the tube tip location 4 (31%) times and failed to mention it 9 (69%) times.	When clinical questions are included in requests for imaging, radiology reports are more likely to answer clinical question
Aubin et al, 2010^33^	Not reported	When the indications for a study included a request for annotations of vertebral arteries (VA), and a definition of VA anomaly, each radiologist described VA (100%)	When clinical questions are included in request for imaging, radiology reports are more likely to answer clinical question

### Reporting time

The impact of clinical information on radiologist reporting time was investigated in two studies.^16^, ^24^ Sarwar et al^16^ reported a 6% decrease in interpretation time when additional clinical information was available. Cooperstein et al^24^ noted only a slight increase in reporting time when clinical information was available. The results relevant to the reporting time outcome measure have been further summarised in Table 4.

## Discussion

The majority of included studies support the notion that clinical information has a positive effect on the reporting process. Studies demonstrated improved interpretation accuracy, clinical relevance and reporting confidence. The addition of clinical information was found not to substantially affect reporting time. These findings were based on studies of moderate quality, with a median quality and risk of bias assessment score of 4 out of 9.^7^ Studies deemed to be of lower quality failed to perform appropriate statistical analysis to demonstrate a statistically significant effect.

These results are in keeping with Loy & Irwig's^4^ systematic review which concluded that clinical information improved interpretation accuracy. Our review provides an updated synthesis of literature to include studies published since Loy & Irwig's^4^ 2004 review, including five on cross‐sectional imaging (e.g. MRI, CT and ultrasound). This review also provides a broader scope of the effect of clinical information on reporting, beyond looking at accuracy alone.

One of the studies investigated the impact of the timing of when clinical information is introduced. Berbaum et al^20^ found that the provision of clinical information at the time of interpretation has a positive effect on radiologist perception, whilst providing this information after interpretation was of no benefit. This study supports the notion that educating referrers to provide quality clinical information to radiologists would result in a greater benefit in reporting outcomes, than radiologists correlating findings with patient notes.

Other studies, which were outside the scope of this review, have investigated the effect of prevalence expectation on diagnostic performance of radiologists. Littlefair et al's^34^ study demonstrates that prior expectations can impact diagnostic efficacy, whereby increased prevalence expectations influence radiologists to assign a false‐positive outcome to a normal image. Although this finding highlights that provision of clinical information can lead to overcalling, the variables tested were extreme and not necessarily reflective of clinical practice. Littlefair et al^34^ recommended referral criteria for those requesting, which is also an outcome of our review.

Another study by Littlefair et al^35^ also discusses the topic of overcalling. Whilst this study focused on the influence of expectation of abnormality and prior knowledge of the outcome, it also indicates that highly specific clinical information can significantly improve location sensitivity. In other words, when specific clinical information is provided to the radiologist prior to image interpretation, the accuracy and clinical relevance of their report can be enhanced.

Our study was limited by the number of eligible studies specific to the research question. Whilst 21 articles were deemed eligible for inclusion, not all of these studies solely focused on the effect of clinical information on the radiology report. Similarly, the broad range of publication dates of included studies may be perceived as a limitation. We found this difficult to restrict as there was no existing review on the effects of clinical information on all aspects of reporting. However, the broad range of publication dates may demonstrate the issue of inadequate clinical information communicated to radiologists has persisted over several decades.

The rationale of three of the most recently published included studies^14^, ^15^, ^30^ may highlight an issue with the quality of clinical information currently being received by radiologists. Doshi et al's^13^ utilisation of patient questionnaires to evaluate the effect on the completeness of clinical information suggests there is a lack of useful clinical information in requests to enable confident reporting. The fact that information provided by patients on the day of their CT scan increased radiologists’ confidence in their findings indicates that useful clinical information was missing in requests. Lacson et al^15^ recognised the limitation of requests but investigated the usefulness of other supplemental sources of information, namely the EHR. Maizlin & Somers^14^ sought to address the shortfall a different way again, by demonstrating that extra clinical information added by radiographers had a positive impact on the resultant report. These three examples could be described as workarounds, defined as solutions which health professionals (and others) use to avoid hindrances to efficiency and achieve improvements in workflow.^36^ The interventions implemented in these studies suggest the perceived communication between referrer and radiologist needs improvement.

Whilst many of the included studies shared similar elements of design, it was clear there was no gold standard or standardisation of requirements for clinical information. This made results difficult to compare, as many studies relied on the expert opinion of radiologists to determine whether clinical information was deemed important or useful when reporting. This measurement of usefulness of clinical information varied across studies, as radiologists taking part in studies would have had different training, skills and specialisations.

In contrast, both Cooperstein et al^24^ and Qureishi et al^31^ specified the type of clinical information required from the requesting clinician. Cooperstein et al's^24^ criteria for clinical information were generalised and could be used for any examination, and the results of the study demonstrated no significant effect on reporting. However, Qureishi et al's^31^ departmental guidelines for clinical information required in requests were specific to CT temporal bone examinations. The guidelines specifically identified key information to be provided in requests and were found to demonstrate a positive impact on clinical relevance and confidence in reporting. As there are more than two decades between the publications, it is possible that the technological advancements in CT and its increased utility^37^ have prompted further investigation into the topic of clinical information to assist with reporting. This idea is supported by Leslie et al^18^ who found the importance of clinical information to increase with the complexity of imaging, due to the greater volume of images produced and the greater list of differential diagnoses. Subsequently, the role clinical information plays is accentuated. It is possible that a lack of clinical information would be a risk factor for missed diagnoses and reduced confidence in incidental findings. In such cases, adequate clinical information may assist radiologists to contextualise incidental findings and subsequently add value to the report.

Given the findings of this review regarding clinical information and its effect on the accuracy, confidence, clinical relevance and timeliness of reporting, Qureishi et al's^31^ study provided evidence for a novel intervention for improving clinical information provided, in the form of departmental guidelines. The guidelines served as a criteria standard, as they outlined recommendations for specific elements of clinical information useful for reporting a particular examination. Criteria standards have been previously used to educate and change behaviours of referrers when requesting by Gunderman et al^38^ who sought to educate referrers on Health Care Financing Administration regulations to improve billing efficiency. This intervention improved compliance with the regulations. Subsequently, the frequency of inadequate clinical information on requests was decreased by approximately two‐thirds.

It is clear the lack of clinical information in requests is an issue affecting reporting quality. One of the possible causes for this may be a lack of awareness or education of referring clinicians on what constitutes relevant clinical information. It may be in the best interests of radiologists to seek to educate referrers on the effect of clinical information on diagnostic performance, including the rationale behind providing high‐quality clinical information.^38^ This need for further education is reflected in a recent study by Glenn‐Cox et al,^39^ who identified that Australian junior doctors do not feel confident to request medical imaging tests accurately. With 66% of Australian junior doctors surveyed claiming to request imaging once a day or more frequently,^39^ it is expected that development of criteria standards for clinical information when requesting medical imaging would be advantageous in improving the quality of the radiology report.

## Conclusion

The findings of this review indicate that clinical information communicated to the radiologist has a positive impact on the radiology report. These results are relevant to the main consumers of medical imaging, those being referrers and by extension their patients. These results are also relevant to radiologists, as they demonstrate the potential improvement that the communication of clinical information can have on the quality of reporting. It is in the best interests of radiologists to communicate the importance of clinical information for reporting via the creation of criteria standards to guide the requesting practices of medical imaging referrers.
